# Iatrogenic Spinal Epidural Hematoma Associated with Intracranial Hypotension

**DOI:** 10.7759/cureus.4171

**Published:** 2019-03-04

**Authors:** Sameer S Ali, Allison E Shaw, Martin Oselkin, Ilya Bragin

**Affiliations:** 1 Neurology, Veterans Affairs Connecticut Healthcare System, West Haven, USA; 2 Neurology, St. Luke's University Health Network, Bethlehem, USA; 3 Radiology, St. Luke's University Health Network, Bethlehem, USA; 4 Neurology, St. Lukes University Health Network, Bethlehem, USA

**Keywords:** cervical epidural, pain management, spinal epidural hematoma, csf leak, targeted blood patch, intracranial hypotension

## Abstract

Epidural steroid injections (ESIs) are one of the few modalities currently in use for treating chronic spinal pain. There are two approaches: interlaminar ESIs and transforaminal ESIs. Complications arising from either approach are rare, but one such complication is cerebrospinal fluid (CSF) leak leading to intracranial hypotension. Even rarer is the development of iatrogenic spinal epidural hematoma in the context of the injections. Interestingly, an association with intracranial hypotension and spinal epidural hematoma has yet to be established. Even the characteristics of an iatrogenic spinal epidural hematoma are not well defined as there are different theories of how this develops and whether we are dealing with arterial or venous blood. Our case is unique as it appears our patient had developed not one, but both clinical symptoms supportive of intracranial hypotension from a CSF leak induced iatrogenically from a cervical epidural injection and imaging demonstrated thoracic-level spinal epidural hematoma. It is unclear whether the injection directly led to the spinal leak causing the intracranial hypotension, which then brought on the formation of the hematoma or if the injection led to both intracranial hypotension and hematoma formation independent of each other. From a clinical practice standpoint, given our case suggests the hematoma was concomitantly associated with intracranial hypotension, and the possibility exists that the hematoma may have formed in the context of the intracranial hypotension, then targeted blood patches may need to be done with greater urgency to preventing hematoma formation. Further studies are needed involving clotting factors comparing arterial and venous blood. It is also puzzling why the epidural blood from the hematoma did not clot the leak. This concomitancy deserves further attention and may lead to changes in how we manage cervical epidural injection patients who are found to have CSF leak and a spinal epidural hematoma.

## Introduction

Spinal pain is one of the more common causes of chronic pain in the United States. According to the 2012 National Health Interview Survey, 54.5% of adults in the United States have a musculoskeletal pain disorder [1]. Various modalities are available for treatment including pharmacotherapy, physical therapy, surgery, neuromodulation, and interventional pain management options including epidural steroid injections (ESIs). ESIs remain a common nonsurgical procedure performed for the treatment of chronic spinal pain when conservative measures have failed [2].

The two utilized approaches to cervical epidural injections include interlaminar ESI (ILESI) and transforaminal ESI (TFESI) approaches. During TFESI, the steroid is injected into the neuroforamen between vertebrae by the exiting nerve root, while in an ILESI, the steroid is injected into the dorsal epidural space between the lamina of the vertebra. Although complications following ESI remain rare, these can include vomiting, systemic hypotension, neck pain at the injection site, nerve root injury, infection, abscess, and dural puncture, causing intracranial hypotension [3].

Direct needle trauma may lead to serious complications including spinal hematomas and cerebrospinal fluid (CSF) leaks. A spinal epidural hematoma is a collection of blood in the potential space known as the epidural space located between the dura and the walls of the vertebral canal, located just outside the dural sac containing the spinal cord and CSF. According to Halim et al., the most common presentation of a spinal epidural hematoma is acute onset pain and radicular symptoms that mimic disc herniation [4]. CSF leaks are the extravasation of CSF from the subarachnoid space causing intracranial hypotension. This can cause debilitating postural headaches that worsen upon sitting and or standing. Additional clinical symptoms may include neck pain, nausea, vomiting, vertigo, and visual and hearing disturbances. They most commonly occur from CSF leaks at a single or along multiple points along the neuroaxis. Various imaging modalities can be useful in identifying the specific location of the CSF leak. Magnetic resonance imaging (MRI), radioisotope cisternography, computed tomography (CT) myelography, and dynamic CT myelograms have all been utilized to locate the specific area of CSF leakage. CT scanning may show subdural collection, dural venous sinus distention, and acquired tonsillar ectopia, while MRI will commonly show pachymeningeal thickening and enhancement, a cranial subdural collection, and cerebellar tonsillar herniation [5]. Being able to identify the site of CSF leakage is helpful in targeting the specific area with a therapeutic blood patch and while a targeted approach is best [6]. if a site cannot be identified, a blindly placed lumbar blood patch may be attempted. Conservative measures are the mainstay of therapy, and they include bed rest and caffeine while a minority of patients need further intervention.

There have been rare cases of cervical epidural injections causing epidural hematomas. However, an association with intracranial hypotension or decreases in intracranial pressure has not been established. Herein, we present a case of intracranial hypotension following a seemingly uncomplicated cervical ESI causing a cervical/upper thoracic CSF leak and an associated thoracic epidural hematoma.

## Case presentation

A 28-year-old woman with a history of chronic neck pain in the context of myofascial pain, previously undiagnosed mild Chiari type-1 malformation, and multilevel cervical disc herniations most prominent at C4-C5, mild at C3-C4 and C5-C6 presented to the hospital with a severe postural headache for the past month. She denied significant headache history. At baseline, she had pain that radiated from the neck into both shoulders with no symptoms in her extremities. She had been treated by a pain management consultant who had performed a cervical interlaminar epidural injection which yielded no pain relief and a right-sided C4, C5, C6 medial branch block for suspected facet arthropathy six months later, again with no relief. Meanwhile, she was taking diclofenac gel, cyclobenzaprine, and tramadol as needed with limited relief from the tramadol.

She decided to seek out another pain practice and received another two rounds of cervical interlaminar epidurals nearly 11 and 12 months after the initial injections, respectively. Immediately after the 11th-month epidural, she began to develop a throbbing headache at the cervico-occipital junction that went away a few hours later with rest and acetaminophen but then returned four to five days later in an extremely intense manner. Her headache was associated with lightheadedness, nausea, and vomiting. She proceeded to have multiple visits to the emergency department (ED) over the last few days with nothing helping, including narcotics. She has tried multiple over-the-counter medications as well. On the third ED visit, three weeks after the last injection, a more detailed history revealed a clear postural component. Given the severity of her headache and associated symptoms, she was admitted for further care.

**Figure 1:**
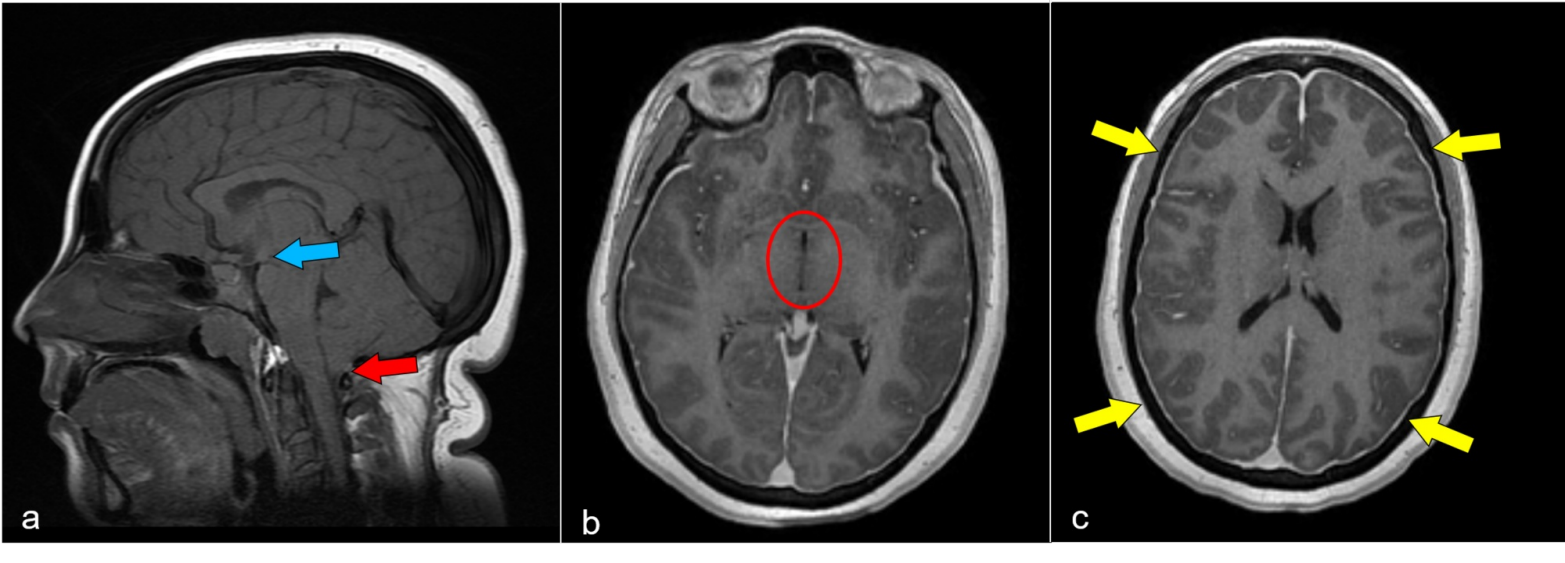
Intracranial hypotension Initial pre-operative MRI. Multiple characteristic features of intracranial hypotension are present in this patient. The sagittal non-contrast T1-weighted image (a) demonstrates mild tonsillar decent (red arrow) and crowding at the foramen magnum as well as a decreased mamillopontine distance (blue arrow). Post-contrast axial T1-weighted images (b,c) demonstrate a slit-like third ventricle (circle) and pachymeningeal enhancement (yellow arrows). MRI: magnetic resonance imaging

She had a repeat head CT in the ED, which was stable. The primary service had the patient sent down for a fluoroscopically guided lumbar puncture to rule out infectious and inflammatory pathology. The CSF analysis results were unremarkable, and the official reported opening pressure was within the reference range at 14 cm. The findings of the detailed neurological examination remained nonfocal and consistent throughout the hospitalization. She did have some tenderness of bilateral splenius capitis and levator scapulae muscles in her neck more so than the bilateral frontalis and temporalis muscles in her head. An MRI of her brain showed a mild Chiari I malformation with mild pachymeningeal enhancement and effacement of the prepontine cistern suggestive of intracranial hypotension (Figure 1).

**Figure 2:**
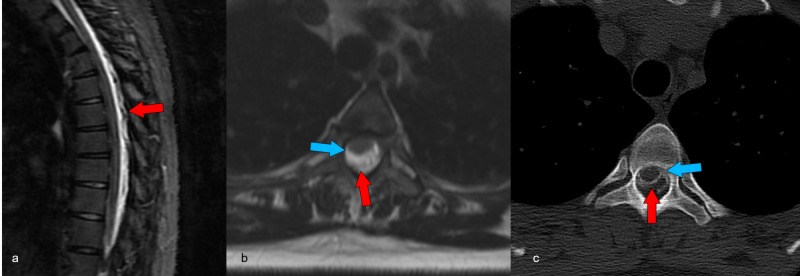
Spinal epidural hemorrhage The sagittal T2-weighted MRI image reveals (a) posterior epidural fluid collection with loculations compatible with a mixture of CSF leak and epidural hematoma (red arrows). A corresponding axial T2-weighted image (b) at T3–T4 also demonstrates this finding. There is effacement and displacement of the thecal sac and cord anteriorly (blue arrow). Axial CT myelogram image at T3–4 (c) shows epidural contrast with superimposed epidural hemorrhage causing effacement and displacement of the thecal sac and cord anteriorly as seen on MRI. MRI: magnetic resonance imaging, CSF: cerebrospinal fluid, CT: computed tomography

A cervical MRI showed no acute changes, and an MRI of the thoracic spine was initially read as normal, but a later review uncovered a thoracic epidural hematoma (Figure 2).

**Figure 3:**
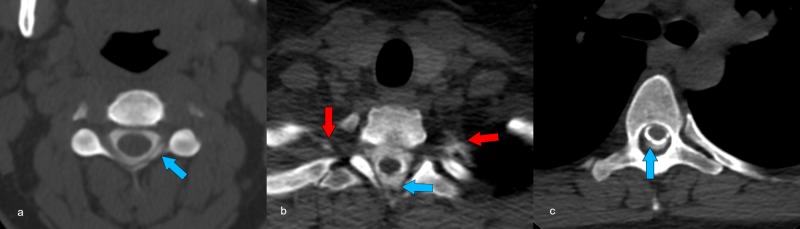
Pre-operative CT myelogram Multiple axial CT images acquired after intrathecal injection of contrast under fluoroscopy demonstrate epidural contrast (blue arrows) spanning from the C3–4 level (a) to the T7 level (c). Contrast flow was so brisk that it is already seen diffusing into the intercostal space (red arrows) at the T2–3 level. Abbreviation: CT, computed tomography.

She did not worsen, given the lumbar puncture, and there was a modest improvement in symptoms after the initiation of intravenous fluids, caffeine pills, metoclopramide, valproate, and ketorolac. However, on approximately the fourth day of hospitalization, her condition worsened, although not to the original severity on the first day when she could not sit up given the severity of the postural component. On the fifth day, she was able to walk to the bathroom and in the halls (briefly). Given her improvement, plans were made for an empiric outpatient blood patch which was performed at the L2/L3 level. She had no improvement, and the next day, she presented to the ED again where she was discharged after the headache went down to a three of 10 on the visual analog pain scale with pharmacotherapy. She developed lower back tightness and mild hip pain from the lumbar patch and continued to have headaches. Three days later, she had a CT myelogram done that showed extensive CSF leak into the epidural space in the cervical and upper thoracic spine (Figure 3).

Two weeks after the myelogram, she had targeted, multi-level CT-guided interlaminar epidural blood patch injections at the C5-C6, C7-T1, and T2-T3 with subsequent complete resolution of her postural headaches.

## Discussion

Spinal epidural hematomas are thought to be from many etiologies. Iatrogenic factors for spinal epidural hematomas include coagulopathy from anticoagulant therapy, antiplatelet therapy, and spinal puncture (e.g., epidural anesthesia, epidural catheter insertion, diagnostic lumbar puncture, and epidural injections). Non-iatrogenic factors for this condition include metabolically or genetically induced coagulopathy, trauma, and pregnancy. Conflicting literature exists on whether the bleeding is arterial or venous [7]. Spontaneous spinal epidural hematoma is reported to be rare [4,8], and those iatrogenically induced from epidural injections appear to be even rarer. According to Caputo et al., millions of ESIs have been completed since 1960. However, there have been no more than 15 reported cases of epidural hematoma related to ESIs and nearly all had been taking anticoagulants at the time of injection [9]. 

Increased intrathoracic and intraabdominal pressure may lead to brief increases in intravenous pressure as blood pooling occurs in the valveless, thin-walled epidural veins, leading to venous rupture [10]. While this may be a valid explanation for subacute or chronic hematomas of the lumbar region, it does not hold true in the cervical region as venous pressure is low in the epidural veins in this region, even lower than the intrathecal pressure [4]. Rapid spinal cord compression could favor an arterial bleed, and a theory suggests the source of the bleeding is free anastomotic arteries that run in the epidural space and connect with the radicular arteries. This is supported by the fact that most spontaneous cervical epidural hematomas occur in the C6/C7 region. This spinal segment is a highly mobile segment of the vertebral column, and certain movements at this region or level can stretch the anastomotic arteries beyond their limits, causing them to rupture [11]. 

Our patient had intracranial hypotension concomitantly with a spinal epidural hematoma. Although intracranial subdural hematoma is a well-known complication of intracranial hypotension, spinal epidural hematoma has been only described once in the setting of intracranial hypotension [8]. We believe we have a similar case with an important difference: the former case involved a spontaneous spinal epidural hematoma, and our case appears to be iatrogenic secondary to a seemingly uncomplicated cervical ESI causing a dural leak. Although we do not exactly know when her hematoma developed, her intracranial hypotension manifested symptoms on the same day of her last cervical epidural injection. Perhaps the dura was susceptible to tear from repeated polytrauma from the prior cervical epidural injections. We cannot exclude the possibility that she may have already had a low-grade chronic but asymptomatic CSF leak, and that the most recent cervical epidural injection added to this leak, and then she developed clinical symptoms of intracranial hypotension.

Epidural blood patches have been the mainstay treatment for intractable patients with a known dural CSF leak dating back as far as 1955 [12-14]. Most epidural blood patches are performed at the lumbar level [15]. Spinal cord compression following a cervical epidural blood patch remains the primary concern with respect to treating cervical CSF leak. There is class II level of evidence that cervical epidural blood patches are safe and effective in relieving postural headaches secondary to CSF leak [15]. While fluoroscopy is used for lumbar blood patches, CT guidance is recommended for cervical blood patches to minimize the risk of spinal cord damage [6]. A localized, multi-level cervical blood patch was ultimately performed in this case after the lumbar epidural blood patched failed.

## Conclusions

Here we described a rare case of spinal epidural hematoma occurring in the setting of iatrogenic CSF leak and intracranial hypotension. The relationship between the epidural blood and CSF leak remains unclear. The existing epidural blood does not necessarily “patch” the leak. However, a targeted blood patch with venous blood can be an effective treatment option. The index of suspicion for CSF leaks should be high if patients note orthostatic headaches, even if there are potentially conflicting data, including normal opening pressure, and prompt targeted therapy should be offered. It’s unclear whether the injection directly led to the spinal leak causing the intracranial hypotension which then brought on the formation of the hematoma or if the injection led to both intracranial hypotension and hematoma formation independent of each other. Clinically, given that our case suggests the hematoma was concomitantly associated with intracranial hypotension, and the possibility exists that the hematoma may have formed in the context of the intracranial hypotension, there may need to be a greater urgency in using targeted blood patches to prevent spinal epidural hematoma formation. Further studies are warranted involving clotting factors comparing arterial and venous blood. The reason why the epidural blood from the hematoma did not clot the leak presents a clinical puzzle. This concomitancy deserves further attention and may lead to changes in the management of cervical epidural injection patients who are found to have both CSF leak and a spinal epidural hematoma.
